# Comprehensive data compilation on the mechanical properties of refractory high-entropy alloys

**DOI:** 10.1016/j.dib.2018.10.071

**Published:** 2018-10-26

**Authors:** J.-P. Couzinié, O.N. Senkov, D.B. Miracle, G. Dirras

**Affiliations:** a Université Paris Est, ICMPE (UMR 7182) CNRS-UPEC, 2-8 rue Henri Dunant, 94320 Thiais, France; b Air Force Research Laboratory, Materials and Manufacturing Directorate, Wright-Patterson AFB, OH 45433, USA; c Université Paris 13, LSPM (UPR 3407) CNRS, 99 avenue J.B. Clément, 93430 Villetaneuse, France

## Abstract

This data article presents the compilation of mechanical properties for 122 refractory high entropy alloys (RHEAs) and refractory complex concentrated alloys (RCCAs) reported in the period from 2010 to the end of January 2018. The data sheet gives alloy composition, type of microstructures and the metallurgical states in which the properties are measured. Data such as the computed alloy mass density, the type of mechanical loadings to which they are subjected and the corresponding macroscopic mechanical properties, such as the yield stress, are made available as a function of the testing temperature. For practical use, the data are tabulated and some are also graphically presented, allowing at a glance to access relevant information for this attractive category of RHEAs and RCCAs.

**Specifications table**

**Table:** 

Subject area	Materials Science
More specific subject area	*Refractory high-entropy alloys (RHEAs) and refractory complex concentrated alloys (RCCAs)*
Type of data	*Table, figures*
How data was acquired	*Compilation of data from available literature. Data extracted from studies on 122 alloys reported in the period from 2010 to January 2018*
Data format	*Analyzed*
Experimental factors	*Data compilation from available literature. Data sheet contains about 54 references.*
Experimental Features	*Extensive Data compilation. Alloys’ mass densities and Young modulus were computed using the rule of mixtures (ROM) for the different reported alloy compositions.*
Data source location	*From the literature, as well as the authors’ calculations. References are given in the corresponding sections.*
Data accessibility	*Data are with the article*
Related research article	*Direct submission. Most relevant research article: Senkov, Oleg; Miracle, Daniel; Chaput, Kevin; Couzinie, Jean-Philippe,*
	*Development and Exploration of Refractory High Entropy Alloys – A Review, Journal of Materials Research, 33 (19), (2018), 3092–3128*, https://doi.org/10.1557/jmr.2018.153[1]

**Value of the data**

• The comprehensive data compilation provides up-to-date mechanical properties of RHEAs and RCCAs tested under uniaxial loading on the basis of published reports from 2010 through the end of January 2018.
• The dataset contains pertinent references, readily accessible to all researchers.
• Processed data may be used to evaluate the potential of RHEAs and RCCAs as possible structural materials.
• The data compilation can be used as a primary tool and as a guidance for further development of RHEAs and RCCAs.
• This data compilation can enable machine learning and data analytics methods to extract insights and trends not available from individual studies, thus accelerating the development of these alloys.

## 1 Data

Refractory High Entropy Alloys (RHEAs) and Refractory Complex Concentrated Alloys (RCCAs) are attractive materials and promising candidates for structural high temperature applications. Deriving from a new alloying design strategy, RHEAs contain five or more elements with concentration between 5 and 35 at% and RCCAs expand this vast range of new alloys even further by including three or more principal elements and expanding the concentrations of these elements beyond 35% [1]. Further, RHEAs are sometimes considered to be only single-phase, disordered solid solution alloys, while RCCAs can have any number of phases and can also include ordered, intermetallic phases. The presented database is a compilation of the mechanical properties of RHEAs and RCCAs from a large number of studies published during the 2010-January 2018 period. Each row in Table 1 corresponds to one mechanical test for an alloy composition in an experimentally characterized metallurgical condition. The data are gathered in a table compiling all the published results such that it could be graphically represented and analyzed afterward [2]. The table also provides the alloy densities calculated in this work using rule of mixtures (ROM), as well as Young׳s moduli for single-phase alloys calculated using ROM.

**Table 1:** RHEAs and RCCAs for which mechanical tests are reported in literature. Each line represents the result of a test on a specific alloy composition. The experimental Young modulus is given in brackets in the adequate column. Values appearing in brackets in the yield strength column correspond to the fracture stress without plastic deformation See text for explanations [57], [58], [59], [60].

## 2 Experimental design, materials, and methods

The presented data sheet is a compilation of essential data on RHEAs and RCCAs. All RHEAs and RCCAs reported in the literature through the end of January 2018 crystallize with at least one phase with body centered cubic (BCC) structure. The results of 340 mechanical tests on 122 compositions are listed and then partially synthesized in graphical form for better visualization.

Table 1 of the data sheet illustrates the collected data from published studies so far [3], [4], [5], [6], [7], [8], [9], [10], [11], [12], [13], [14], [15], [16], [17], [18], [19], [20], [21], [22], [23], [24], [25], [26], [27], [28], [29], [30], [31], [32], [33], [34], [35], [36], [37], [38], [39], [40], [41], [42], [43], [44], [45], [46], [47], [48], [49], [50], [51], [52], [53], [54], [55], [56], for all the RHEAs / RCCAs:

• the *alloy composition*. Alloying elements are classified by alphabetic order and the subscripts indicate atom mole fraction. A subscript of 1 is implied if none is shown.
• the *metallurgical state* of each tested alloy: non-equilibrium state such as-cast state, or optimized state via homogenization and annealing, thermally-processed conditions.
• the *phase content* present in the initial testing condition. From the mechanical properties point of view, it appears crucial, whether an alloy consists of a single phase, or of several phases.
• the *type of mechanical test*: tension or compression. Only mechanical tests with strain rates less than or equal to 10^−3^ s^−1^ are considered here.
• the *testing temperature*.
• The *experimental Young modulus*, when reported.
• the *yield strength σ_Y_*.

The *density* of each of the 122 compositions have been calculated on the basis of Rule of Mixtures (ROM) (Eq. 1):

(1) $$\rho_{\mathrm{alloy}}=\frac{\sum_{i=1}^{N}c_{i}A_{i}}{\sum_{i=1}^{N}c_{i}M_{i}}$$

Where *c*_i_ is the atomic fraction of element *i* in the alloy; A_*i*_ and Μ_*i*_ are the molar mass and molar volume of element *i* at room temperature. The *specific strength* is important for some structural applications. Therefore, such an important feature for structural part design, when available, is also listed in Table 1.

The *Young modulus* have also been estimated using ROM for single phase solid solutions (Eq. 2):

(2) $$E_{\mathrm{alloy}}=\sum_{i=1}^{N}x_{i}E_{i}$$

With x_*i*_ and E_*i*_ are the atomic fraction and the room temperature Young modulus of the alloy element *i*. Young modulus calculated from *ab initio* methods or determined experimentally are also provided in the table.

Acronyms used in Table 1 represent:

RT: Room Temperature
ROM: Rule of Mixtures
AC: As-Cast
A: Annealed
HIP: Hot Isostatic Pressured
CR: Cold Rolled
SPS: Spark Plasma Sintering
SPD: Severe Plastic Deformation
T: Tension (tensile test)
C: Compression (compressive test)

**Fig. 1:**
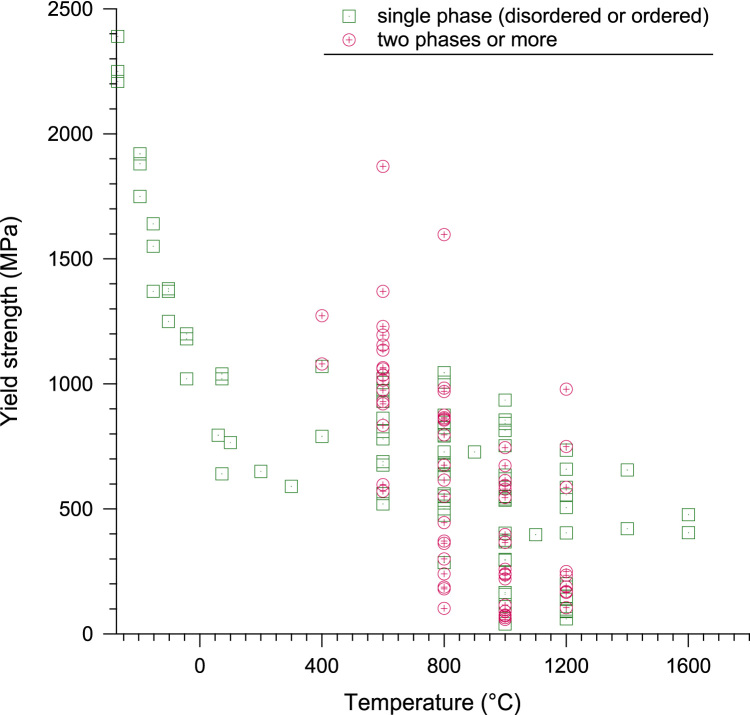
Evolution of yield strength with temperature in the −268.8 °C–1600 °C range. For the sake of clarity all the collected data at room temperature have been excluded of this figure.

**Fig. 2:**
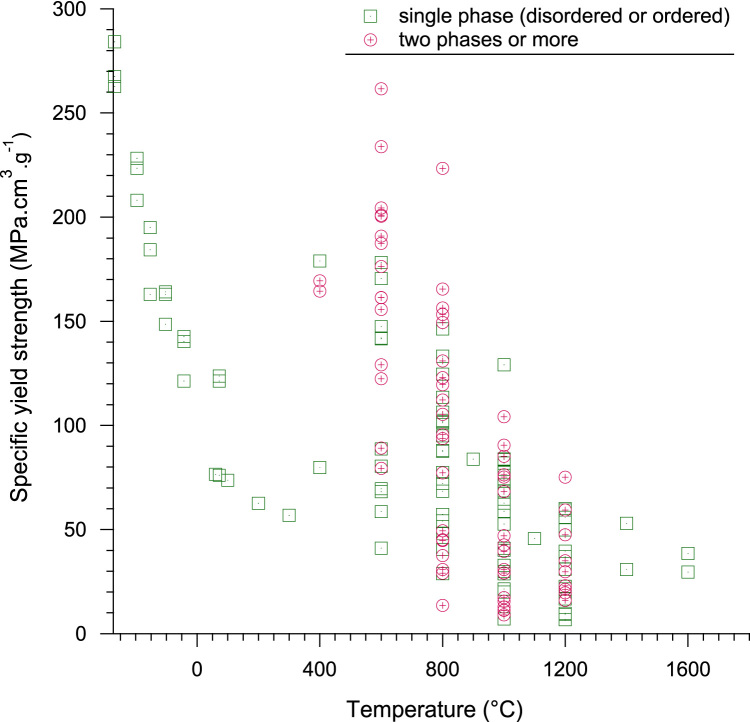
Evolution of specific yield strength with temperature in the −268.8 °C–1600 °C range. For the sake of clarity all the collected data at room temperature have been excluded of this figure.

It can be seen from Table 1 of the data sheet that RHEAs and RCCAs have been studied over a wide temperature range between −268.8 °C (4.2 K) and 1600 °C (1873 K). For quick access and reading, a quantitative representation of the compiled data is illustrated in Fig. 1, Fig. 2. This shows the evolution of yield strength and specific yield strength with temperature for a single phase or multiphase, multi-component alloys whatever the equilibrium condition/alloy processing.

**Fig. 3:**
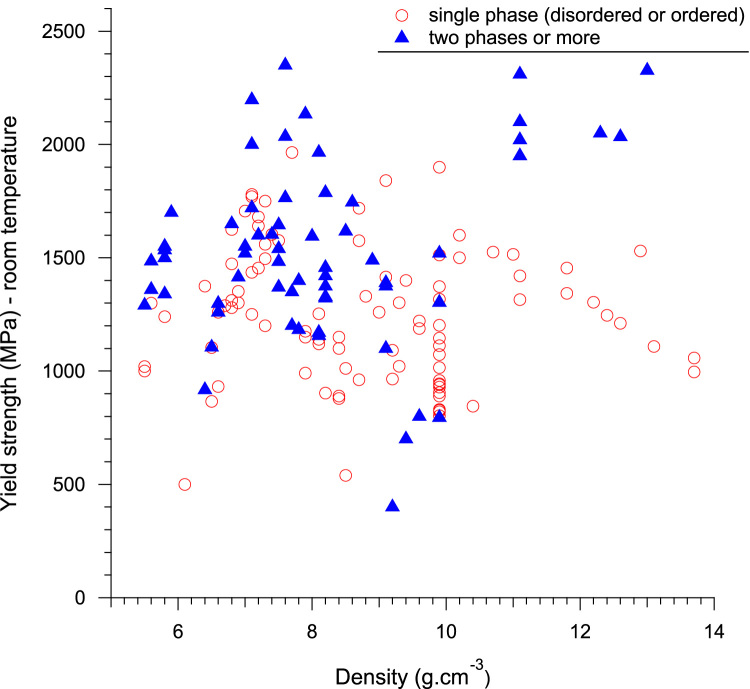
Evolution of yield strength of RHEAs and RCCAs with alloy density at room temperature. Single and multi-phase alloys are distinguished.

**Fig. 4:**
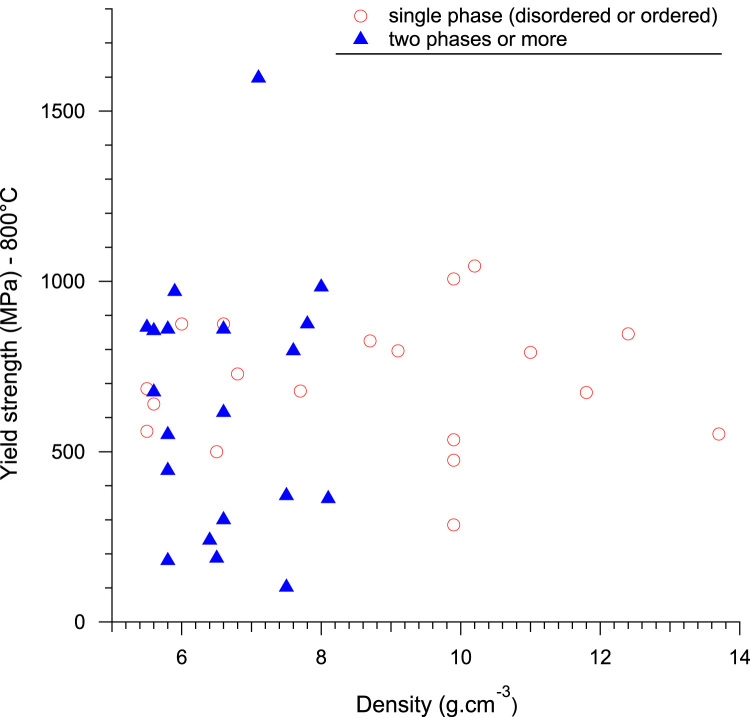
Evolution of yield strength of RHEAs and RCCAs with alloy density at 800 °C. Single and multi-phase alloys are distinguished.

The data have been processed in order to directly visualize the evolution of mechanical properties with density, which could be very useful in the research for material solutions for applications at a given temperature. Fig. 3, Fig. 4 display the evolution of yield stress with alloy density for the different multi-component at room temperature and 800 °C, respectively.

## References

[1] Senkov O.N., Miracle D.B., Chaput K.J., Couzinie J.-P. (2018). Development and exploration of refractory high entropy alloys – a review. J. Mater. Res..

[2] Gorsse S., Miracle D.B., Senkov O.N. (2017). Mapping the world of complex concentrated alloys. Acta Mater..

[3] Chen S.Y., Yang X., Dahmen K.A., Liaw P.K., Zhang Y. (2014). Microstructures and crackling noise of AlxNbTiMoV high entropy alloys. Entropy.

[4] X. Yang, Y. Zhang, P.K. Liaw, Microstructure and Compressive Properties of NbTiVTaAlx High Entropy Alloys, in: C.M. Wang, C.J. Peng (Eds.), Materials Science Forum, 2012: pp. 292–298.

[5] Senkov O.N., Jensen J.K., Pilchak A.L., Miracle D.B., Fraser H.L. (2018). Compositional variation effects on the microstructure and properties of a refractory high-entropy superalloy AlMo0.5NbTa0.5TiZr. Mater. Des..

[6] Qiao D., Jiang H., Chang X., Lu Y., Li T. (2017). Microstructure and mechanical properties of VTaTiMoAlx refractory high entropy alloys. Mater. Sci. Forum.

[7] Lin C.-M., Juan C.-C., Chang C.-H., Tsai C.-W., Yeh J.-W. (2015). Effect of Al addition on mechanical properties and microstructure of refractory AlxHfNbTaTiZr alloys. J. Alloy. Compd..

[8] Senkov O.N., Woodward C., Miracle D.B. (2014). Microstructure and properties of aluminum-containing refractory high-entropy alloys. JOM.

[9] Senkov O.N., Senkova S.V., Woodward C. (2014). Effect of aluminum on the microstructure and properties of two refractory high-entropy alloys. Acta Mater..

[10] Stepanov N.D., Yurchenko N.Y., Panina E.S., Tikhonovsky M.A., Zherebtsov S.V. (2017). Precipitation-strengthened refractory Al0.5CrNbTi2V0.5 high entropy alloy. Mater. Lett..

[11] Stepanov N.D., Yurchenko N.Y., Skibin D.V., Tikhonovsky M.A., Salishchev G.A. (2015). Structure and mechanical properties of the AlCrxNbTiV (x=0, 0.5, 1, 1.5) high entropy alloys. J. Alloy. Compd..

[12] Chen H., Kauffmann A., Gorr B., Schliephake D., Seemueller C., Wagner J.N., Christ H.-J., Heilmaier M. (2016). Microstructure and mechanical properties at elevated temperatures of a new Al-containing refractory high-entropy alloy Nb-Mo-Cr-Ti-Al. J. Alloy. Compd..

[13] Chen H., Kauffmann A., Laube S., Choi I.C., Schwaiger R., Lichtenberg K., Müller F., Gorr B., Christ H.-J., Heilmaier M. (2017). Contribution of lattice distortion to solid solution strengthening in a series of refractory high entropy alloys. Metall. Trans. A—Phys. Metall. Mater. Sci..

[14] Senkov O.N., Isheim D., Seidman D.N., Pilchak A.L. (2016). Development of a refractory high entropy superalloy. Entropy.

[15] Stepanov N.D., Shaysultanov D.G., Salishchev G.A., Tikhonovsky M.A. (2015). Structure and mechanical properties of a light-weight AlNbTiV high entropy alloy. Mater. Lett..

[16] Yurchenko N.Y., Stepanov N.D., Zherebtsov S.V., Tikhonovsky M.A., Salishchev G.A. (2017). Structure and mechanical properties of B2 ordered refractory AlNbTiVZrx (x=0–1.5) high-entropy alloys. Mater. Sci. Eng. A-Struct. Mater. Prop. Microstruct. Process..

[17] Guo N.N., Wang L., Luo L.S., Li X.Z., Chen R.R., Su Y.Q., Guo J.J., Fu H.Z. (2016). Microstructure and mechanical properties of in-situ MC-carbide particulates-reinforced refractory high-entropy Mo0.5NbHf0.5ZrTi matrix alloy composite. Intermetallics.

[18] Zhang M., Zhou X., Li J. (2017). Microstructure and mechanical properties of a refractory CoCrMoNbTi high-entropy alloy. J. Mater. Eng. Perform..

[19] Fazakas E., Zadorozhnyy V., Varga L.K., Inoue A., Louzguine-Luzgin D.V., Tian F., Vitos L. (2014). Experimental and theoretical study of Ti20Zr20Hf20Nb20X20 (X = V or Cr) refractory high-entropy alloys. Int. J. Refract. Met. Hard Mater..

[20] Senkov O.N., Woodward C.F. (2011). Microstructure and properties of a refractory NbCrMo0.5Ta0.5TiZr alloy. Mater. Sci. Eng. A-Struct. Mater. Prop. Microstruct. Process..

[21] Senkov O.N., Senkova S.V., Miracle D.B., Woodward C. (2013). Mechanical properties of low-density, refractory multi-principal element alloys of the Cr-Nb-Ti-V-Zr system. Mater. Sci. Eng. A-Struct. Mater. Prop. Microstruct. Process..

[22] Waseem O.A., Lee J., Lee H.M., Ryu H.J. (2017). The effect of Ti on the sintering and mechanical properties of refractory high-entropy alloy TixWTaVCr fabricated via spark plasma sintering for fusion plasma-facing materials. Mater. Chem. Phys..

[23] Feuerbacher M., Heidelmann M., Thomas C. (2015). Plastic deformation properties of Zr-Nb-Ti-Ta-Hf high-entropy alloys. Philos. Mag..

[24] Guo N.N., Wang L., Luo L.S., Li X.Z., Chen R.R., Su Y.Q., Guo J.J., Fu H.Z. (2016). Microstructure and mechanical properties of refractory high entropy (Mo0.5NbHf0.5ZrTi)(BCC)/M5Si3 in-situ compound. J. Alloy. Compd..

[25] Sheikh S., Shafeie S., Hu Q., Ahlstrom J., Persson C., Vesely J., Zyka J., Klement U., Guo S. (2016). Alloy design for intrinsically ductile refractory high-entropy alloys. J. Appl. Phys..

[26] Podolskiy A.V., Tabachnikova E.D., Voloschuk V.V., Gorban V.F., Krapivka N.A., Firstov S.A. (2018). Mechanical properties and thermally activated plasticity of the Ti30Zr25Hf15Nb20Ta10 high entropy alloy at temperatures 4.2–350 K. Mater. Sci. Eng. A-Struct. Mater. Prop. Microstruct. Process..

[27] Juan C.-C., Tseng K.-K., Hsu W.-L., Tsai M.-H., Tsai C.-W., Lin C.-M., Chen S.-K., Lin S.-J., Yeh J.-W. (2016). Solution strengthening of ductile refractory HfMoxNbTaTiZr high-entropy alloys. Mater. Lett..

[28] Liu Y., Zhang Y., Zhang H., Wang N., Chen X., Zhang H., Li Y. (2017). Microstructure and mechanical properties of refractory HfMo0.5NbTiV0.5Six high-entropy composites. J. Alloy. Compd..

[29] Juan C.-C., Tsai M.-H., Tsai C.-W., Lin C.-M., Wang W.-R., Yang C.-C., Chen S.-K., Lin S.-J., Yeh J.-W. (2015). Enhanced mechanical properties of HfMoTaTiZr and HfMoNbTaTiZr refractory high-entropy alloys. Intermetallics.

[30] Guo N.N., Wang L., Luo L.S., Li X.Z., Su Y.Q., Guo J.J., Fu H.Z. (2015). Microstructure and mechanical properties of refractory MoNbHfZrTi high-entropy alloy. Mater. Des..

[31] Lilensten L., Couzinie J.-P., Bourgon J., Perriere L., Dirras G., Prima F., Guillot I. (2017). Design and tensile properties of a bcc Ti-rich high-entropy alloy with transformation-induced plasticity. Mater. Res. Lett..

[32] Zhang Y., Liu Y., Li Y., Chen X., Zhang H. (2016). Microstructure and mechanical properties of a refractory HfNbTiVSi0.5 high-entropy alloy composite. Mater. Lett..

[33] Zhang Y., Liu Y., Li Y., Chen X., Zhang H. (2016). Microstructure and mechanical properties of a new refractory HfNbSi0.5TiVZr high entropy alloy. Mater. Sci. Forum.

[34] Schuh B., Voelker B., Todt J., Schell N., Perriere L., Li J., Couzinie J.-P., Hohenwarter A. (2018). Thermodynamic instability of a nanocrystalline, single-phase TiZrNbHfTa alloy and its impact on the mechanical properties. Acta Mater..

[35] Senkov O.N., Semiatin S.L. (2015). Microstructure and properties of a refractory high-entropy alloy after cold working. J. Alloy. Compd..

[36] Juan C.-C., Tsai M.-H., Tsai C.-W., Hsu W.-L., Lin C.-M., Chen S.-K., Lin S.-J., Yeh J.-W. (2016). Simultaneously increasing the strength and ductility of a refractory high-entropy alloy via grain refining. Mater. Lett..

[37] Lilensten L., Couzinie J.-P., Perriere L., Hocini A., Keller C., Dirras G., Guillot I. (2018). Study of a bcc multi-principal element alloy: tensile and simple shear properties and underlying deformation mechanisms. Acta Mater..

[38] Senkov O.N., Scott J.M., Senkova S.V., Miracle D.B., Woodward C.F. (2011). Microstructure and room temperature properties of a high-entropy TaNbHfZrTi alloy. J. Alloy. Compd..

[39] Couzinie J.-P., Lilensten L., Champion Y., Dirras G., Perriere L., Guillot I. (2015). On the room temperature deformation mechanisms of a TiZrHfNbTa refractory high-entropy alloy. Mater. Sci. Eng. A-Struct. Mater. Prop. Microstruct. Process..

[40] Dirras G., Couque H., Lilensten L., Heczel A., Tingaud D., Couzinie J.-P., Perriere L., Gubicza J., Guillot I. (2016). Mechanical behavior and microstructure of Ti20Hf20Zr20Ta20Nb20 high-entropy alloy loaded under quasi-static and dynamic compression conditions. Mater. Charact..

[41] Dirras G., Lilensten L., Djemia P., Laurent-Brocq M., Tingaud D., Couzinie J.-P., Perriere L., Chauveau T., Guillot I. (2016). Elastic and plastic properties of as-cast equimolar TiHfZrTaNb high-entropy alloy. Mater. Sci. Eng. A-Struct. Mater. Prop. Microstruct. Process..

[42] Senkov O.N., Scott J.M., Senkova S.V., Meisenkothen F., Miracle D.B., Woodward C.F. (2012). Microstructure and elevated temperature properties of a refractory TaNbHfZrTi alloy. J. Mater. Sci..

[43] Maiti S., Steurer W. (2016). Structural-disorder and its effect on mechanical properties in single-phase TaNbHfZr high-entropy alloy. Acta Mater..

[44] Wu Y.D., Cai Y.H., Wang T., Si J.J., Zhu J., Wang Y.D., Hui X.D. (2014). A refractory Hf25Nb25Ti25Zr25 high-entropy alloy with excellent structural stability and tensile properties. Mater. Lett..

[45] Huang H., Wu Y., He J., Wang H., Liu X., An K., Wu W., Lu Z. (2017). Phase-transformation ductilization of brittle high-entropy alloys via metastability engineering. Adv. Mater..

[46] Wu Y.D., Cai Y.H., Chen X.H., Wang T., Si J.J., Wang L., Wang Y.D., Hui X.D. (2015). Phase composition and solid solution strengthening effect in TiZrNbMoV high-entropy alloys. Mater. Des..

[47] Han Z.D., Luan H.W., Liu X., Chen N., Li X.Y., Shao Y., Yao K.F. (2017). Microstructures and mechanical properties of TixNbMoTaW refractory high-entropy alloys. Mater. Sci. Eng. A-Struct. Mater. Prop. Microstruct. Process..

[48] Yao H.W., Qiao J.W., Hawk J.A., Zhou H.F., Chen M.W., Gao M.C. (2017). Mechanical properties of refractory high-entropy alloys: experiments and modeling. J. Alloy. Compd..

[49] Han Z.D., Chen N., Zhao S.F., Fan L.W., Yang G.N., Shao Y., Yao K.F. (2017). Effect of Ti additions on mechanical properties of NbMoTaW and VNbMoTaW refractory high entropy alloys. Intermetallics..

[50] Wang S.-P., Xu J. (2017). TiZrNbTaMo high-entropy alloy designed for orthopedic implants: as-cast microstructure and mechanical properties. Mater. Sci. Eng. C-Mater. Biol. Appl..

[51] Todai M., Nagase T., Hori T., Matsugaki A., Sekita A., Nakano T. (2017). Novel TiNbTaZrMo high-entropy alloys for metallic biomaterials. Scr. Mater..

[52] Yao H., Qiao J.-W., Gao M.C., Hawk J.A., Ma S.-G., Zhou H. (2016). MoNbTaV medium-entropy alloy. Entropy.

[53] Kang B., Lee J., Ryu H.J., Hong S.H. (2018). Ultra-high strength WNbMoTaV high-entropy alloys with fine grain structure fabricated by powder metallurgical process. Mater. Sci. Eng. A-Struct. Mater. Prop. Microstruct. Process..

[54] Senkov O.N., Wilks G.B., Scott J.M., Miracle D.B. (2011). Mechanical properties of Nb25Mo25Ta25W25 and V20Nb20Mo20Ta20W20 refractory high entropy alloys. Intermetallics.

[55] Zhang Y., Yang X., Liaw P.K. (2012). Alloy design and properties optimization of high-entropy alloys. JOM.

[56] Yao H.W., Qiao J.W., Gao M.C., Hawk J.A., Ma S.G., Zhou H.F., Zhang Y. (2016). NbTaV-(Ti,W) refractory high-entropy alloys: experiments and modeling. Mater. Sci. Eng. A-Struct. Mater. Prop. Microstruct. Process..

[57] Cao P., Ni X., Tian F., Varga L.K., Vitos L. (2015). Ab initio study of AlxMoNbTiV high-entropy alloys. J. Phys.-Condens. Matter.

[58] Zheng S.-M., Feng W.-Q., Wang S.-Q. (2018). Elastic properties of high entropy alloys by MaxEnt approach. Comput. Mater. Sci..

[59] Tian F., Varga L.K., Chen N., Shen J., Vitos L. (2014). Ab initio design of elastically isotropic TiZrNbMoVx high-entropy alloys. J. Alloy. Compd..

[60] Tian L.-Y., Wang G., Harris J.S., Irving D.L., Zhao J., Vitos L. (2017). Alloying effect on the elastic properties of refractory high-entropy alloys. Mater. Des..

